# *Helicobacter pylori* Induces Disturbances in Gastric Mucosal Akt Activation through Inducible Nitric Oxide Synthase-Dependent S-Nitrosylation: Effect of Ghrelin

**DOI:** 10.5402/2011/308727

**Published:** 2010-11-04

**Authors:** Bronislaw L. SLomiany, Amalia Slomiany

**Affiliations:** Research Center, C875 University of Medicine and Dentistry of New Jersey Dental School, 110 Bergen Street, P.O. Box 1709, Newark, NJ 07103-2400, USA

## Abstract

Gastric mucosal inflammatory response to *H. pylori *and its key virulence factor, lipopolysaccharide (LPS), are characterized by a massive rise in apoptosis and the disturbances in NO signaling pathways. Here, we report that *H. pylori* LPS-induced enhancement in the mucosal inducible nitric oxide synthase (iNOS) was associated with the suppression in Akt kinase activity and the impairment in constitutive nitric oxide synthase (cNOS) phosphorylation. Further, we demonstrate that the LPS effect on Akt inactivation, manifested in the kinase protein S-nitrosylation and a decrease in its phosphorylation at Ser^473^, was susceptible to suppression by iNOS inhibition. Moreover, the countering effect of hormone, ghrelin, on the LPS-induced changes in Akt activity was reflected in the loss in Akt S-nitrosylation and the increase in its phosphorylation at Ser^473^, as well as cNOS activation through phosphorylation. Our findings demonstrate that up-regulation in iNOS with *H. pylori *infection leads to Akt inactivation through S-nitrosylation that exerts the detrimental effect on the processes of cNOS activation through phosphorylation. We also report that ghrelin protection against *H. pylori*-induced disturbances is manifested in a marked increase in Akt activity and evoked by a decrease in the kinase S-nitrosylation and the increase in its phosphorylation at Ser^473^.

## 1. Introduction

Ghrelin, an endogenous ligand for growth-hormone secretagogue receptor type 1a (GHSR1a), initially recognized for its role in the regulation of food intake and energy homeostasis, has emerged recently as an important factor in the control of local inflammations, gastroprotection, and the modulation of gastric mucosal inflammatory responses to *H. pylori* infection [1–5]. The signaling mechanism that underlies GHSR1a stimulation by ghrelin involves the activation of heterotrimeric G protein-dependent pathways that result in a multiple downstream network of protein kinases, including Src/Akt kinase pathway implicated in the regulation of nitric oxide synthase (NOS) system responsible for NO production [5–7].

The physiological and pathophysiological implications of NO depend on its local concentration, the type of NOS isozyme involved in NO generation, substrate availability, and the enzyme compartmentalization with respect to protein target [8, 9]. A low level of NO generated by membrane-associated Ca^2+^/calmoduline-dependent constitutive (c) cNOS appears to access a pool of substrates that are of importance to the maintenance of normal physiological functions which include the regulation of cell-signaling events associated with apoptogenic signal propagation [5, 8, 10, 11]. On the other hand, the high level of NO generated by more distant cytosolic Ca^2+^/calmodulin-independent inducible (i) iNOS in response to proinflammatory cytokines and bacterial LPS has been implicated in host response to sepsis and endotoxemia [9, 12]. However, sustained iNOS activation associated with persistence of inflammatory stimulus is also known to have cytotoxic consequences reflected in transcriptional derangements and the induction of apoptosis [1, 11, 12]. Therefore, the disturbances in NO production associated with *H. pylori *colonization and reflected in continual activation of iNOS and the suppression of cNOS isozyme systems [13, 14] may be of major consequence in defining the extent of gastric mucosal inflammatory involvement.

The mechanism underlying cNOS activity is controlled by a complex set of pre- and posttranslational factors that affect dynamics of its subcellular targeting and the activity by exposing the enzyme to fatty acid modification through N-myristoylation and thiopalmitoylation, interaction with regulatory cofactors, and the protein phosphorylation at the critical Ser^1179^ with the involvement of protein kinase Akt [5–7, 15–17]. This serine/threonine kinase, also known as protein kinase B (PKB) or Akt/PKB, is a central player in the regulation of apoptosis, cell cycle and metabolic pathways, and signal transduction pathways activated by growth factors and insulin as well as ghrelin [5, 6, 12, 17, 18]. Activation of Akt in response to insulin or ghrelin occurs downstream of phosphoinositide 3-kinase (PI3K) and involves the generation of the lipid second messenger phosphatidylinositol-3,4,5-triphosphate, which accumulates in the plasma membrane and serves as a recognition site for the N-terminal PH (pleckstrin homology) domain of Akt [18, 19]. The induced conformational changes in Akt result in the exposure to phosphorylation within the activation (A) loop at Thr^308^ and of Ser^473^ located within the HM (hydrophobic motif) region of the C-terminal domain of Akt [18]. These two sites of phosphorylation are apparently necessary for full activation of Akt [12, 18].

The accumulating evidence, furthermore, indicates that the activity of Akt may be also regulated through S-nitrosylation at the kinase cysteine residues [12, 20]. Indeed, S-nitrosylation of Akt by the exogenous NO donors and the NO produced by iNOS has been implicated in conferring insulin resistance, and Akt S-nitrosylation was reported to be associated with the reduced kinase activity in muscle cells of genetically obese, diabetic (db/db) mice [20, 21]. Moreover, protein modification through targeted S-nitrosylation at the critical cysteine residues is gaining recognition as an important posttranslational event, that like posttranslational modification through phosphorylation regulates a variety of signal transduction events involving NO [5, 8–10, 17, 22].

Gastric ghrelin has been identified as an important regulator of NOS system responsible for NO production [6, 17, 23], and we have shown recently that the countering effect of ghrelin on *H. pylori *LPS-induced gastric mucosal cell apoptosis was reflected in the increase in cNOS activity [5]. As protein kinase Akt plays a central role in a rapid posttranslational cNOS activation through phosphorylation [6, 15, 16], in this study we investigated further the nature of the impairment in cNOS activation induced in gastric mucosal cells by *H. pylori *LPS**. **Our data revealed that the impairment in gastric mucosal cNOS phosphorylation with the LPS results from iNOS-induced suppression in Akt activity through S-nitrosylation. We further showed that the countering effect of ghrelin was reflected in a marked increase in Akt phosphorylation and a decrease in its protein S-nitrosylation.

## 2. Materials and Methods

### 2.1. Gastric Mucosal Cell Incubation

 The mucosal cells, collected by scraping the mucosa of freshly dissected rat stomachs with a blunt spatula, were suspended in five volumes of ice-cold Dulbecco's modified (Gibco) Eagle's minimal essential medium (DMEM), supplemented with fungizone (50 *μ*g/ml), penicillin (50 U/ml), streptomycin (50 *μ*g/ml), and 10% fetal calf serum, gently dispersed by trituration with a syringe, and settled by centrifugation [5]. Following rinsing, the cells were resuspended in the medium to a concentration of 2 × 10^7^ cell/ml, transferred in 1 ml aliquots to DMEM in culture dishes, and incubated under 95% O_2_ : 5% CO_2_ atmosphere at 37°C for 16 h in the presence of 0–200 ng/ml of *H. pylori *LPS [5]. In the experiments evaluating the effect of ghrelin (rat, Sigma), cNOS inhibitor, L-NAME, iNOS inhibitor, 1400W, Akt inhibitor, SH-5 (Calbiochem), and ascorbate (Sigma), the cells were first preincubated for 30 min with the indicated dose of the agent or vehicle before the addition of the LPS. The viability of cell preparations before and during the experimentation, assessed by Trypan blue dye exclusion assay [24], was greater than 97%.

### 2.2. Helicobacter pylori Lipopolysaccharide

*H. pylori *used for LPS preparation was cultured from clinical isolates obtained from ATCC no. 4350 [14]. The bacterium was homogenized with liquid phenol-chloroform-petroleum ether and centrifuged, and the LPS contained in the supernatant was precipitated with water, washed with 80% phenol solution and dried with ether. The dry residue was dissolved in a small volume of water at 45°C and centrifuged at 100,000 × g for 4 h, and the resulting LPS sediment subjected to lyophilization.

### 2.3. cNOS and iNOS Activity Assay

 Nitric oxide synthase activities of cNOS and iNOS enzymes in the gastric mucosal cells were measured by monitoring the conversion of L-[^3^H] arginine to L-[^3^H] citrulline using NOS-detect kit (Stratagene). The cells from the control and experimental treatments were homogenized in a sample buffer containing either 10 mM EDTA (for Ca^2+^-independent iNOS) or 6 mM CaCl_2_ (for Ca^2+^-depenedent cNOS) and centrifuged. The aliquots of the resulting supernatant were incubated for 30 min at 25°C in the presence of 50 *μ*Ci/ml of L-[^3^H] arginine, 10 mM NAPDH, 5 *μ*M tetrahydrobiopterin, and 50 mM Tis-HCl buffer, pH 7.4, in a final volume of 250 *μ*l. Following addition of stop buffer and Dowex-50 W (Na^+^) resin, the mixtures were transferred to spin cups and centrifuged and the formed L-[^3^H]citrulline contained in the flow through was quantified by scintillation counting.

### 2.4. Akt Activity Assay

 The kinase activity of Akt in gastric mucosal cells was measured with the Akt Activity Kit (Calbiochem) by quantifying phosphorylation of a biotinylated peptide substrate (GRPRTSSFAEG). The cells were lysed in lysis buffer (20 mM Tris-HCl, pH 7.4, 150 mM NaCl, 10% glycerol, 1% Triton X-100, 1% deoxycholate, 2 mM EDTA, 1 mM sodium orthovanadate, 1 mM PAF, and 1 mM NaF), containing protease inhibitor cocktail (Sigma), at 4°C for 30 min, centrifuged at 14,000 × g for 15 min, and immunoprecipitated with anti-Akt antibody for 1 h at 4°C. Protein A/G agarose beads were then added for additional 1 h, and the immune complex was recovered by centrifugation and thoroughly washed with lysis buffer. The agarose beads were then suspended for 30 min at room temperature in the kinase assay buffer and centrifuged, and the supernatants were used for the Akt activity assay by following the manufacturer's instruction.

### 2.5. Akt Phosphorylation Assay

 Assessment of the phosphorylation status of Akt in gastric mucosal cells was carried out using Akt (pThr^308^) and Akt (pSer^473^) ELISA kits (Calbiochem). The mucosal cells were lysed on ice for 30 min in lysis buffer (10 mM Tris-HCl, pH 7.4, 100 mM NaCl, 1 mM EDTA, 1 mM EGTA, 1 mM NaF, 2 mM sodium orthovanadate, 1% Triton X-100, 10% glycerol, 0.1% SDS, 0.5% deoxycholate, and 1 mM PMSF), containing protease inhibitor cocktail and centrifuged at 14,000 × g for 15 min. The supernatants diluted (1 : 10) in standard diluent buffer were pipetted in 100 *μ*l aliquots into wells containing immobilized capture antibody, and after washing, the complex was reacted with antibody specific for Akt (pThr^308^) or Akt (pSer^473^). After washing, the retained complex was labeled with horseradish peroxidase and probed with TMB reagent for spectrophotometric quantification at 450 nm.

### 2.6. Akt Protein S-Nitrosylation Assay

 A biotin switch procedure was employed to assess Akt enzyme protein S-nitrosylation [25, 26]. The gastric mucosal cells were treated with iNOS inhibitor, 1400W (30 *μ*M) or ghrelin (0.5 *μ*g/ml), and incubated for 16 h in the presence of 100 ng/ml of *H. pylori *LPS. Following centrifugation at 500 × g for 5 min, the recovered cells were lysed in 0.2 ml of HEN lysis buffer (250 mM HEPES, 1 mM EDTA, 0.1 mM neocuprin, and pH 7.7), and the unnitrosylated thiol groups were blocked with S-methyl methanethiosulfonate reagent at 50°C for 20 min [26]. The proteins were precipitated with acetone, resuspended in 0.2 ml of HEN buffer containing 1% SDS, and subjected to targeted nitrothiol group reduction with sodium ascorbate (100 mM). The free thiols were then labeled with biotin, and the biotinylated proteins were recovered on streptavidin beads. The formed streptavidin bead-protein complex was washed with neutralization buffer, and the bound proteins were dissociated from streptavidin beads with 50 *μ*l of elution buffer (20 mM HEPES, 100 mM NaCl, 1 mM EDTA, and pH 7.7) containing 1% 2-mercaptoethanol [26]. The obtained proteins were then analyzed by Western blotting.

### 2.7. Immunoblotting Analysis

The mucosal cells from the control and experimental treatments were collected by centrifugation and resuspended for 30 min in ice-cold lysis buffer (20 mM Tris-HCl, pH 7.4, 150 mM NaCl, 10% glycerol, 1% Triton X-100, 2 mM EDTA, 1 mM sodium orthovanadate, 4 mM sodium pyrophosphate, 1 mM PMSF, and 1 mM NaF), containing 1 *μ*g/ml leupeptin and 1 *μ*g/ml pepstatin [5]. Following brief sonication, the lysates were centrifuged at 12,000 g for 10 min, and the supernatants were subjected to protein determination using BCA protein assay kit (Pierce). The samples, including those subjected to biotin switch procedure, were then resuspended in loading buffer, boiled for 5 min, and subjected to SDS-PAGE using 40 *μ*g protein/lane. The separated proteins were transferred onto nitrocellulose membranes, blocked for 1 h with 5% skim milk in Tris-buffered Tween (20 mM Tris-HCl, pH 7.4, 150 mM NaCl, and 0.1% Tween-20), and probed with the antibody against phosphorylated protein at 4°C for 16 h. After 1 h incubation with the horseradish peroxidase-conjugated secondary antibody, the phosphorylated proteins were revealed using an enhanced chemiluminescence. Membranes were stripped by incubation in 1 M Tris-HCl (pH 6.8), 10% SDS, and 10 mM dithiotreitol for 30 min at 55°C and reprobed with antibody against total cNOS or Akt. Immunoblotting was performed using specific antibodies directed against cNOS, phospho-cNOS (Ser^1179^) and Akt, phospho-Akt (Ser^473^) (Calbiochem).

### 2.8. Data Analysis

 All experiments were carried out using duplicate sampling, and the results are expressed as means ± SD. Analysis of variance (ANOVA) followed by nonparametric Kruskal-Wallis test was used to determine significance, and the significance level was set at *P* < .05.

## 3. Results

**Figure 1:**
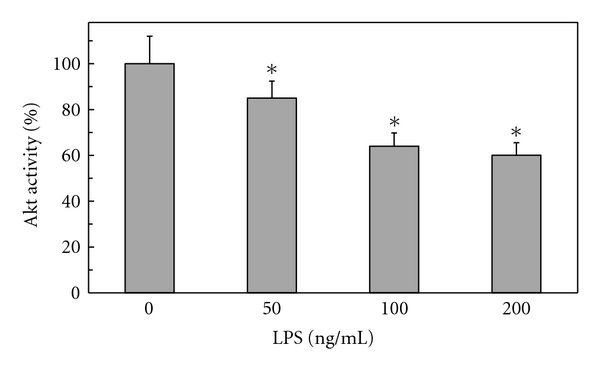
Effect of *H. pylori *LPS on Akt kinase activity in rat gastric mucosal cells. The cells were treated with the indicated concentrations of the LPS and incubated for 16 h. Values represent the means ± SD of five experiments. *P < .05 compared with that of control (LPS-0).

**Figure 2:**
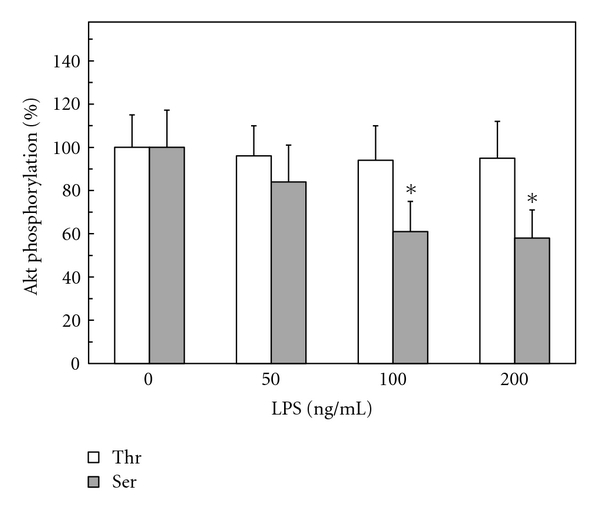
Effect of *H. pylori *LPS on Akt kinase threonine (Thr^308^) and serine (Ser^473^) phosphorylation in rat gastric mucosal cells. The cells were treated with the indicated concentrations of the LPS and incubated for 16 h. *P < .05 compared with that of control (LPS-0).

To further understand the modulatory role ghrelin on the disturbances in NOS system associated with gastric mucosal inflammatory responses to *H. pylori *infection, we used rat gastric mucosal cells and examined the effect of *H. pylori *key virulence factor, LPS, on the activity of a serine/threonine kinase, Akt. We found that the LPS caused a dose-dependent drop in the mucosal cell Akt activity, which at 100 ng/ml LPS decreased by a 36% (Figure 1). Moreover, we demonstrated that the inhibitory effect of the LPS on Akt activity was reflected in a 39% drop in the enzyme Ser^473^ phosphorylation, while phosphorylation of the enzyme protein at Thr^308^ was not affected (Figure 2). 

**Figure 3:**
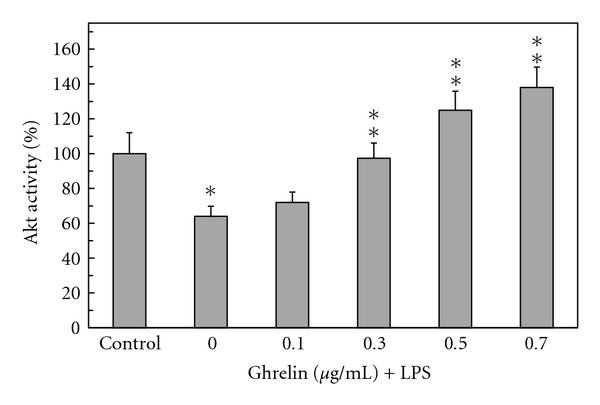
Effect of ghrelin on *H. pylori *LPS-induced changes in gastric mucosal cell Akt kinase activity. The cells, preincubated with the indicated concentrations of ghrelin, were treated with the LPS at 100 ng/ml and incubated for 16 h. *P < .05 compared with that of control. **P < .05 compared with that of LPS alone.

**Figure 4:**
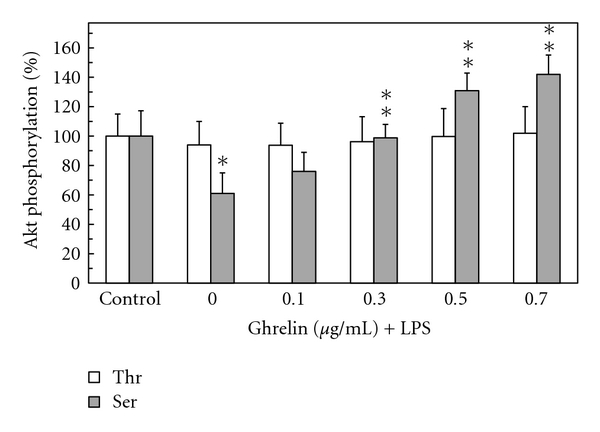
Effect of ghrelin on *H. pylori *LPS-induced changes in gastric mucosal cell Akt kinase threonine (Thr^308^) and serine (Ser^473^) phosphorylation. The cells, preincubated with the indicated concentrations of ghrelin, were treated with the LPS at 100 ng/ml and incubated for 16 h. Values represent the means ± SD of five experiments. *P < .05 compared with that of control. **P < .05 compared with that of LPS alone.

**Figure 5:**
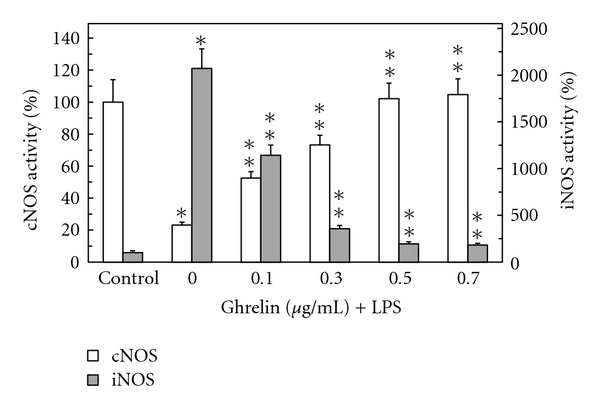
Effect of ghrelin on *H. pylori *LPS-induced expression of cNOS and iNOS activities in gastric mucosal cells. The cells, preincubated with the indicated concentrations of ghrelin, were treated with the LPS at 100 ng/ml and incubated for 16 h. Values represent the means ± SD of five experiments. *P < .05 compared with that of control. **P < .05 compared with that of LPS alone.

Furthermore, we found that preincubation of gastric mucosal cells with ghrelin led to a concentration-dependent suppression of the LPS-induced effect on Akt activity and the extent of its protein phosphorylation on Ser^473^. As a result, the activity of Akt in the presence of 0.5 *μ*g/ml ghrelin increased twofolds over that of the LPS (Figure 3), while the enzyme protein phosphorylation at Ser^473^ showed a 2.1-fold increase (Figure 4). We also established that the LPS at 100 ng/ml elicited a 19.8-fold increase in the mucosal cell iNOS activity, while the cNOS activity showed a 4.3-fold decrease. Preincubation with ghrelin at 0.5 *μ*g/ml resulted in a 77.7% reversal of the LPS inhibitory effect on the mucosal cell cNOS activity as well as produced a 90.2% reduction in the LPS-induced iNOS activity (Figure 5).

**Figure 6:**
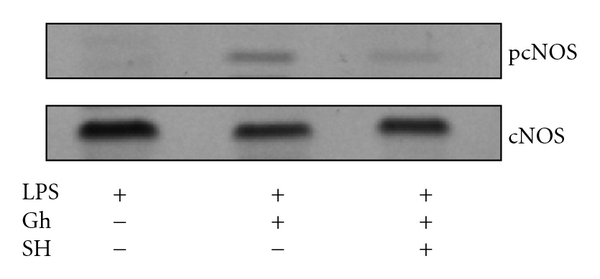
Effect of Akt kinase inhibitor, SH-5 (SH), on ghrelin-induced cNOS phosphorylation in the gastric mucosal cells exposed to *H. pylori *LPS. The cells were treated with ghrelin (Gh) at 0.5 *μ*g/ml or Akt inhibitor, SH-5 (30 *μ*M) + Gh, and incubated for 16 h in the presence of 100 ng/ml LPS. Cell lysates were resolved on SDS-PAGE, transferred to nitrocellulose, and probed with phosphorylation-specific cNOS (pcNOS) antibody, and after stripping reprobed with anti-cNOS antibody. The immunoblots shown are representative of three experiments.

To reveal further insights into the involvement of Akt in the regulation of NOS system, we examined the effect of ghrelin on cNOS activation. As cNOS is known to undergo a rapid posttranslational activation through phosphorylation at Ser^1179^ [5, 6], the mucosal cells prior to the incubation with ghrelin were pretreated with Akt kinase inhibitor, SH-5, and the lysates were probed with antibodies directed against cNOS and phosphorylated cNOS at Ser^1179^ (Figure 6). The results revealed that the LPS-induced suppression in cNOS activity was associated with the inhibition in the enzyme protein phosphorylation, while the reversal of the LPS effect by ghrelin was reflected in a marked increase in the enzyme protein phosphorylation at Ser^1179^. Furthermore, we observed a drop in the ghrelin-induced cNOS phosphorylation in the presence of Akt inhibitor, SH-5 (Figure 6). 

**Figure 7:**
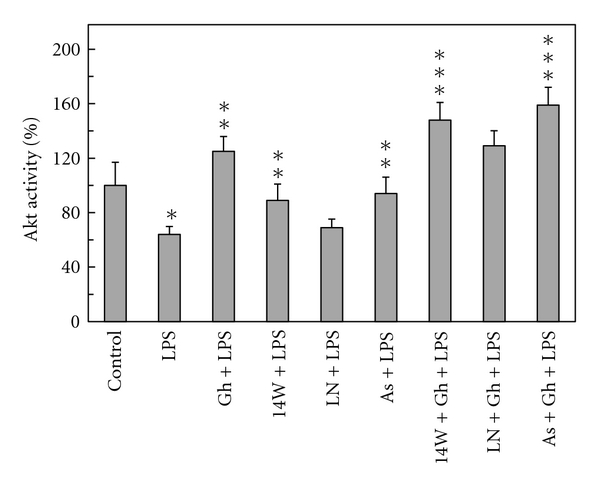
Effect of iNOS inhibitor, 1400W, cNOS inhibitor, L-NAME, and ascorbate on the ghrelin-(Gh-) induced changes in Akt kinase activity in gastric mucosal cell exposed to *H. pylori *LPS. The cells, preincubated with 30 *μ*M 1400W (14W), 300 *μ*M L-NAME (LN), or 300 *μ*M ascorbate (As) were treated with Gh at 0.5 *μ*g/ml and incubated for 16 h in the presence of 100 ng/ml LPS. Values represent the means ± SD of five experiments. *P < .05 compared with that of control. **P < .05 compared with that of LPS alone. ***P < .05 compared with that of Gh *+* LPS.

As the activity of Akt kinase, in addition to the enzyme protein phosphorylation at Thr^308^ and Ser^473^ [12, 18], appears to be regulated through S-nitrosylation at the kinase cysteine residues [12, 20], we next analyzed the effect of cNOS inhibitor, L-NAME, and iNOS inhibitor, 1400W, as well as nitrosothiols reducing agent, ascorbate, on ghrelin-induced changes in gastric mucosal cell Akt activity. As shown in Figure 7, the LPS-induced inhibition in Akt activity was subject to reversal not only by the pretreatment with ghrelin, but also displayed susceptibility to iNOS inhibitor, 1400W, and ascorbate. Moreover, preincubation with these two agents produced amplification in the effect of ghrelin on Akt activity, whereas cNOS inhibitor, L-NAME, had no effect on the extent of the LPS and ghrelin-induced changes in Akt activity (Figure 7). 

**Figure 8:**
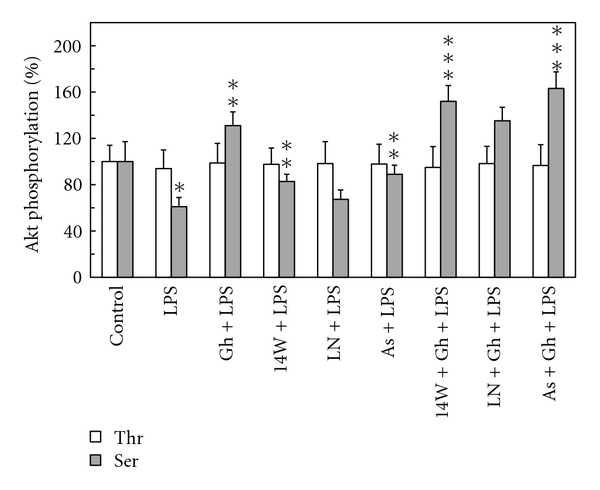
Effect of iNOS inhibitor, 1400W, cNOS inhibitor, L-NAME, and ascorbate on the ghrelin-(Gh-) induced changes in Akt kinase threonine (Thr^308^) and serine (Ser^473^) phosphorylation in gastric mucosal cell exposed to *H. pylori *LPS. The cells, preincubated with 30 *μ*M 1400W (14 W), 300 *μ*M L-NAME (LN), or 300 *μ*M ascorbate (As), were treated with Gh at 0.5 *μ*g/ml and incubated for 16 h in the presence of 100 ng/ml LPS. Values represent the means ± SD of five experiments. *P < .05 compared with that of control. **P < .05 compared with that of LPS alone. ***P < .05 compared with that of Gh *+* LPS.

To gain additional leads as to the mechanism of *H. pylori *LPS-induced suppression in gastric mucosal Akt kinase activation and its reversal by ghrelin, we examined the effect of cNOS and iNOS inhibitors, and nitrosothiols reducing agent, ascorbate, on the Akt enzyme protein phosphorylation at Thr^308^ and Ser^473^. We observed that while Akt phosphorylation at Thr^308^ was not affected by the LPS or ghrelin, the LPS-induced decrease in Akt phosphorylation at Ser^473^ was subject to partial reversal in the presence of iNOS inhibitor, 1400W, and ascorbate, but not the cNOS inhibitor, L-NAME. Furthermore, both ascorbate and 1400W elicited amplification in ghrelin effect on the gastric mucosal Akt Ser^473^ phosphorylation (Figure 8). 

**Figure 9:**
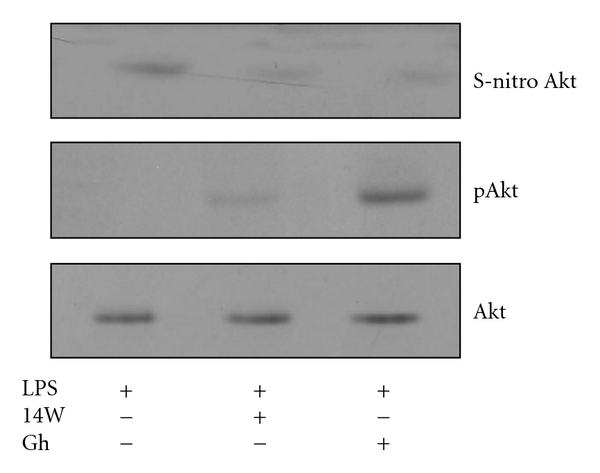
Effect of ghrelin (Gh) on *H. pylori *LPS-induced Akt S-nitrosylation. The gastric mucosal cells were treated with iNOS inhibitor, 1400W (30 *μ*M), or Gh (0.5 *μ*g/ml) and incubated for 16 h in the presence of 100 ng/ml LPS. A portion of the cell lysates were processed by biotin switch procedure for protein S-nitrosylation, along with the reminder of the lysates, resolved on SDS-PAGE, transferred to nitrocellulose and probed with phospho-Akt (Ser^473^) antibody, and after stripping reprobed with anti-Akt antibody. The immunoblots shown are representative of three experiments.

Finally, we examined the dependence of Akt S-nitrosylation on the LPS-induced iNOS activation by the biotin switch method [25, 26]. The gastric mucosal cells were incubated with *H. pylori *LPS or ghrelin + LPS or iNOS inhibitor, 1400W, + LPS, and the lysates following the biotin switch procedure were probed with antibodies directed against phospho-Akt (Ser^473^) and total Akt. Western blot analysis revealed that Akt in the cells exposed to the LPS alone showed a marked increase in the protein S-nitrosylation; the preincubation with iNOS inhibitor, 1400W, led to a pronounced decrease in the LPS-induced Akt S-nitrosylation, whereas the effect of ghrelin was reflected in the loss of Akt S-nitrosylation and the increase in the kinase phosphorylation at Ser^473^ (Figure 9). These data suggest that upregulation in iNOS with *H. pylori *infection and subsequent Akt kinase S-nitrosylation exerts the detrimental effect on the processes dependent on Akt activation, including that of cNOS phosphorylation.

## 4. Discussion

Increase in gastric mucosal proinflammatory cytokine expression, enhancement in epithelial cell apoptosis, and the disturbances in NO signaling pathways are well-recognized features of gastritis associated with *H. pylori *infection in humans as well as characterize mucosal inflammatory responses to *H. pylori *LPS in the animal model of the LPS-induced gastritis [5, 13, 14, 27]. Moreover, the disturbances in NO production associated with *H. pylori *colonization of gastric mucosa and reflected in continual activation of iNOS and the inhibition of cNOS are considered of major consequences in defining the extent of gastric mucosal inflammatory involvement. As serine/threonine kinase Akt plays a central role in the regulation of NOS system [6, 15, 28], in this study we investigated the influence of *H. pylori *on the processes associated with the activation of kinase Akt.

Employing rat gastric mucosal cells exposed to *H. pylori *key virulence factor, LPS, we demonstrated that the LPS-induced drop in cNOS activity and upregulation in iNOS was associated with the suppression in the activity of Akt that was reflected in a decrease in the kinase phosphorylation at Ser^473^, while phosphorylation at the kinase Thr^308^ was not affected. Thus, our findings add further credence to the literature data indicating that of the two phosphorylation sites (Thr^308^ and Ser^473^) required for full activation of Akt; bacterial toxins induce inactivation of Akt that correlates with reduced phosphorylation at the kinase Ser^473^ [21, 29]. Furthermore, we found that preincubation with gastric hormone, ghrelin [1], recognized for its modulatory effect on the inflammatory responses to bacterial infection [2–5], exerted countering effect of the LPS-induced changes in Akt activity and the extent of the kinase phosphorylation on Ser^473^ as well as led to an increase in the cNOS activity and a reduction in the activity of iNOS. We also observed that, in consonance with the documented involvement of Akt in rapid cNOS activation through phosphorylation at Ser^1179^ [5–7], the induced upregulation in cNOS activity by ghrelin was reflected in the increase of enzyme phosphorylation that was susceptible to suppression by Akt inhibitor, SH-5. Since Akt plays a central role in the regulation of NOS system and the signaling mechanism underlying ghrelin action involves the activation of Src/Akt pathway [5, 6, 15, 28], the presented findings point to the role of ghrelin in controlling the extent of gastric mucosal inflammatory consequences of *H. pylori *infection.

Mounting evidence indicates that physiological and pathophysiological implications of NO depend on the type of NOS isozyme involved in NO generation, substrate availability, and the enzyme compartmentalization with respect to signaling target [8, 9]. Moreover, both constitutive and inducible forms of NOS system have been implicated in protein modification through targeted S-nitrosylation at the critical cysteine residues that result in functional alterations [5, 8, 10, 30]. Indeed, S-nitrosylation with the involvement of cNOS has been linked to the apoptogenic signal inhibition and the events of cytosolic phospholipase A_2_ activation, while the NO generated by iNOS has been implicated in S-nitrosylation of proteins involved in insulin signal transduction and the reduced Akt kinase activity in muscle cells of diabetic mouse [5, 17, 20, 21]. Therefore, to gain additional leads into the mechanism of ghrelin suppression of the LPS-induced disturbances in gastric mucosal Akt kinase activation, we examined the effect of NOS inhibitors and nitrosothiols reducing agent, ascorbate, on Akt activity and its protein phosphorylation at the critical Thr^308^ and Ser^473^. We found that, while phosphorylation at Akt Thr^308^ was not affected, the LPS-induced suppression in Akt activity and the extent in its protein phosphorylation at Ser^473^ displayed susceptibility to iNOS inhibitor, 1400W, and ascorbate, but not to cNOS inhibitor, L-NAME. Furthermore, both 1400W and ascorbate elicited amplification in ghrelin effect on Akt activity and its protein phosphorylation at Ser^473^. These data, together with well-known susceptibility of S-nitrosylated proteins to reduction by ascorbate [17, 20, 21, 25, 26], demonstrate that *H. pylori *LPS-induced disturbances in gastric mucosal Akt activity occur with the involvement of iNOS-mediated Akt protein S-nitrosylation that interferes with the kinase activation through phosphorylation at Ser^473^. Moreover, our results suggest that the countering effect of ghrelin on the LPS-induced changes in Akt activity is associated with the loss in Akt S-nitrosylation and the increase in its phosphorylation at Ser^473^. 

The supporting evidence as to the role of ghrelin in the modulation of the gastric mucosal consequences of *H. pylori *interference with Akt activation through S-nitrosylation comes from the results of biotin switch assay. We found that the mucosal cells exposed to incubation with *H. pylori *LPS showed a marked increase in Akt protein S-nitrosylation, whereas the effect of ghrelin on the LPS-induced suppression in Akt activity was reflected in the loss in S-nitrosylation and the increase in the kinase phosphorylation at Ser^473^. Further, Western blot analysis revealed the dependence of Akt S-nitrosylation on the LPS-induced activity of iNOS. We observed that suppression of the iNOS activity with a specific inhibitor, 1400W, led to a drop in Akt S-nitrosylation. These results are indicative of the involvement of iNOS-derived NO in the suppression of Akt activity through S-nitrosylation. Thus, the sustained upregulation in gastric mucosal iNOS, identified patients with gastritis caused by *H. pylori *infection [13, 14, 31, 32], may be of major significance in defining the extent of gastric mucosal inflammatory involvement. 

Taken together, our study demonstrates that the upregulation in iNOS elicited in gastric mucosal cells by *H. pylori *LPS leads to Akt kinase inactivation through S-nitrosylation that exerts the detrimental effect on the processes of cNOS activation through phosphorylation. We also report that ghrelin countering effects against *H. pylori*-induced disturbances are manifested in a marked increase in Akt activity, caused by a decrease in the kinase protein S-nitrosylation and an increase in its phosphorylation at Ser^473^.
